# Driving forces of GPs’ migration in Europe: an exploratory qualitative study

**DOI:** 10.3399/BJGPO.2022.0132

**Published:** 2023-03-08

**Authors:** Marta Velgan, Thierry Vanderheyde, Ruth Kalda, Nele Michels

**Affiliations:** 1 University of Tartu, Tartu, Estonia; 2 Family Medicine and Population Health, University of Antwerp, Antwerp, Belgium; 3 Institute of Family Medicine and Public Health, University of Tartu, Tartu, Estonia

**Keywords:** doctors’ migration, family medicine, general practice, primary health care, qualitative research, recruitment and retention

## Abstract

**Background:**

The shortage of GPs is a worldwide phenomenon, which encourages the migration of GPs and consequently exacerbates the GP shortage. This shortage imposes a threat for the entire healthcare system.

**Aim:**

To explore the driving forces of GPs' migration in Europe and their reasons to stay in the new country, to migrate further, or to return to their home country.

**Design & setting:**

An exploratory, qualitative study of European GPs who have migrated within Europe.

**Method:**

Individual interviews were conducted until data saturation and audio-recordings were transcribed. Thematic analysis was performed using NVivo.

**Results:**

Fifteen interviews with GPs from eight different European countries were conducted. The reasons why European GPs migrate and decide to stay or to leave were grouped under the following three themes: professional development; personal reasons; and the situation in the home country or the organisation of health care. New professional challenges, better working environment, and higher quality training programmes were mentioned as the main reasons for migration. Personal reasons, such as family’s satisfaction with the living environment, closeness to other family members, and financial considerations, seemed to influence the decision to stay or leave the most.

**Conclusion:**

Migration caused by dissatisfaction with the working and living environment is something countries could potentially work on in order to retain their GPs. As some countries in Europe rely more and more on international recruitment to combat the GP shortage, which in turn worsens the situation in other countries, a more unified Europe-wide approach to GP shortage and migration is needed.

## How this fits in

Many countries in Europe struggle with GP shortage. GPs’ migration can be both the reason and the cause for GP shortage. As the migration of healthcare workers occurs usually from low-income countries to high-income countries, it worsens the situation even more in the former. Although doctors’ migration is a well-studied phenomenon, few studies focus on GPs’ migration, especially on GPs’ migration between different European countries. This multi-country study explored the driving forces of GPs’ migration in Europe and the reasons why GPs decide to stay, to return, or migrate further. As such, this study adds additional insight into the reasons behind the GP shortage and retention problems.

## Introduction

The shortage of GPs is a growing problem worldwide.^1–4^ Primary care is considered to be the foundation of the healthcare system by providing accessible and good functioning health care.^3^ In countries where primary care is well-organised, people have better health outcomes and healthcare resources are more equitably distributed among the population.^5^ The low attractiveness of the GP profession, lack of social recognition, negative attitude, difficult working conditions, and lower remuneration compared with other doctors in other specialties are considered to be the leading reasons for the GP shortage.^6^ This crisis is both a cause and a result of GPs’ migration; encouraging the migration of doctors from lower-income to higher-income countries and at the same time causing shortages of doctors in mostly lower-income countries.^4,7–10^

Migration of doctors in the main immigrant-receiving countries, such as the UK, Ireland, the US, Australia, New Zealand and Canada, has been well-studied, while migration of GPs in particular, as well as migration to and from other European countries has only occasionally been researched.^8,10–16^ There are big discrepancies between European countries concerning the number of foreign-trained doctors; ranging from 0.6% of all doctors in Lithuania to 40.8% in Norway and 41.4% in Ireland. Nearly all European Organisation for Economic Cooperation and Development (OECD) countries increasingly rely on recruiting doctors from abroad to fill their shortages.^17^ Why and how people migrate is a complex issue that is being studied worldwide. However, the reasons for the migration of doctors are not so uniform and are constantly changing.^10,16^ The concept of pull and push factors is often being used as a practical framework for identifying and understanding the causes of migration. Push factors encourage the healthcare professional to leave the home country and pull factors facilitate the transfer of a healthcare worker to a new country.^16^ Klein *et al* added ‘plant’ factors to this framework, representing incentives to stay.^18^

As far as the authors know, no multi-country study about doctors, especially GPs, migrating in Europe, has been published yet. As little research focuses on the migration of GPs,^1,2,4,5,19^ this study aims to explore the driving forces of the migration of GPs in Europe as a whole instead of per individualised country and to understand the reasons GPs stay in the new country, return, or migrate further. GP shortage is a worsening problem and understanding the reasons behind GP migration in Europe can give additional insight into the reasons behind retention problems of the GP workforce.

## Method

### Study design and setting

An exploratory qualitative research method was chosen to explore in depth GPs' experiences, beliefs, values and motivations regarding migration.^20–22^

### Participants and recruitment

Through purposive and snowball sampling, using contacts, mailing lists, and online social media platforms (Facebook, Twitter, LinkedIn), emigrant and returned GPs from different World Health Organization (WHO) Europe-countries were recruited to participate in individual interviews. Before the interview, participants completed a questionnaire using SurveyMonkey to collect the following general information: name; contact information; sex; age; the country of origin; the country they have migrated to; the number of years they have lived and worked in the country they migrated to; and whether they have finished residency in family medicine or general practice, and in which country. A total of 31 GPs filled out the questionnaire, of whom 15 GPs agreed to participate. Included GPs were proficient in English. They had migrated after finishing undergraduate medical studies in their home country to start their family medicine or general practice specialisation, or to become a GP abroad, or migrated after having completed family medicine or general practice specialisation in their home country. They had all lived and worked in the new country for at least 1 year.

### Data collection

In-depth individual online interviews were conducted using Zoom. The interview guide (Supplementary Table S1) was developed using data from the literature and was piloted in the first interview, whereafter no changes were made.

### Data analysis

An inductive approach to the analysis was chosen. A thematic analysis following steps set out by Braun and Clarke,^23^ was conducted by the first two authors using NVivo (Release 1). The researchers first familiarised themselves with the data by reading the transcriptions multiple times, whereafter initial codes were generated and themes searched for by each researcher independently. After that, the researchers compared each other’s themes and a common thematic map was created, which was reviewed several times until a unified and mutually satisfying result was reached.

## Results

In total, 15 individual interviews were conducted between March and April 2021 with GPs from eight different countries, lasting 30–60 minutes. Further characteristics of the participants are shown in Supplementary Table S2.

The reasons why GPs migrated, decided to stay, or to leave were grouped under the following three main themes: professional development; personal reasons; and the situation in the home country or the organisation of health care. Supplementary Table S3 shows the themes and sub-themes that were created from the analysis with illustrative quotes. All these themes will be described further in the article, and the different factors can also be found in Supplementary Tables S4 and S5. Participants were assigned an ID number according to the order in which they were interviewed (GP1, GP2, and so on).

### Reasons to migrate

Migration was seen by the participants as both a positive and a negative phenomenon. Migration offered the opportunity to experience life and work abroad, to gain new knowledge and experiences, to meet new people and ideally to take these experiences back home. On the other hand, migration could indicate potential problems and dissatisfaction with the situation in the home country, which could lead people to look for a better working and living environment in another country.

#### Professional development

##### New experiences and challenges

Professional development was brought up as one of the leading reasons for migration. Many GPs felt that their potential as GPs had not been fully reached, or were looking for new professional challenges. Some participants also expressed their interest in experiencing working in another healthcare system.

Many newly qualified GPs had migrated with the aim of gaining more work experience as GPs. They considered working as a GP in their home country to be not as challenging and perceived that GPs faced many constraints, financial or legal, preventing them from realising their full professional potential.

##### Medical education

Better organisation and higher quality of training programmes, higher remuneration, and better supervision were mentioned as reasons some doctors migrated with the aim to specialise in family medicine or general practice abroad. Some participants felt that they were being exploited as trainees in their home country without getting any training and supervision.

### Personal reasons

#### Family

Participants mentioned as a reason to migrate the wish to offer their family a better life and to provide higher quality education for their children, which could give them an advantage in life. The following other personal family related reasons were also mentioned: the wish to live in the countryside; and the wish to move closer to their spouse’s family.

#### Financial reasons

Although participants did not bring financial stability out as the main reason, it was an important determinant in influencing their decision to migrate. Higher remuneration was connected with the wish to provide a better life for their family. According to some participants, this also allowed them to work part-time and therefore achieve a desirable work–life balance.

#### Experience life and work abroad

For some, the simple desire to experience life abroad and to see new places was an important reason for migration.

### Situation in home country

#### Political situation and living environment

Some GPs' migration was driven by their dissatisfaction with the political situation and living environment in their home country. This was related both to the feeling that their efforts and work as GPs were not appreciated, and to the way society functioned in their home country in general. Some mentioned corruption, both outside and inside the healthcare system, which made it difficult for them to achieve their professional goals.

#### Organisation of healthcare system

Participants mentioned the poor organisation of health care, especially the primary healthcare system, in their home country as a reason to migrate. Some participants had been involved in developing the primary healthcare system and the education of GPs in their home country for years and, seeing that nothing had changed, they wanted to experience working in a well-organised healthcare system.

### To stay or to leave

When GPs migrated, some already had the idea of returning home at some point; others migrated without any further plans.

#### Professional development

##### New experiences and challenges

Further professional development, a stimulating working environment with new opportunities not available in their home country, and professional stability were mentioned by the participants as reasons for staying. For some, professional development was the main reason, while others mentioned it as a contributing factor. New career opportunities, such as opening their own practice or continuing developing an existing practice in their home country, motivated some to return. Some experienced plenty of stimulating career opportunities after their return. Others saw migration to a new country as an opportunity for further professional development.

### Personal reasons

#### Family

Family was one of the most important factors in the decision to stay or to leave. Here, the overall degree of satisfaction with their life in the new country, including the education and social circle of their children, seemed to play a major role. Some GPs had decided or planned to return home because they missed family or because of their children’s future. Participants who had not yet made a decision also indicated that family could be a reason for them to go back at some point.

#### Living environment

Satisfaction or dissatisfaction with the living environment was mentioned as a reason to influence participants' decision to stay or to leave. A factor contributing to this satisfaction seems to be the well-established network of social institutions in the countries they migrated to, like social security, taxes, education, and so on. The interaction with local people and the feeling of being welcomed or not also played a role in satisfaction. Some countries are much more family-friendly, making it easier to work part-time and have a better work–life balance. When people had settled in their new environment and started a new life there, it was arduous to leave. The process of migration, especially with a family, was described as difficult and stressful and participants did not want to go through it again.

#### Financial reasons

For some participants, financial considerations played a role in their decision to stay or to leave. Moving back could threaten the newly achieved financial stability and the standard of living that allowed them to have a better work–life balance and future opportunities. Financial investments made in the country they had migrated to made it more complicated to leave. Higher remuneration in another country was mentioned as a reason contributing to further migration. Some participants encountered financial obstacles when planning to return to their home country, which ultimately forced them to migrate further.

#### Experience life and work in a new country

For some participants the main reasons to migrate further was to experience a new kind of adventure and to see how people live and work in other countries.

### Organisation of health care

#### Satisfaction with the organisation of health care

Satisfaction with the organisation and stability of the healthcare system was an important reason for staying in the new country. The way health care, and especially primary health care, was organised, and the working environment of GPs, played a major role in their wish to stay. Another reason preventing leaving was the negative opinion of colleagues and of society in some countries towards migrating GPs. Other participants had seen that not much had changed during their stay abroad, or that the situation of GPs had worsened. The idea of facing the same problems for which they had decided to migrate seemed to discourage some participants from returning home.

#### The way GPs work

Some, on the other hand, found it difficult to adapt to the way GPs worked in the country they migrated to. They were also uncertain about the future of primary health care and the working conditions of GPs if they stayed. These reasons made them decide to leave.

#### Improvement of healthcare system in home country

Although most of the participants seem satisfied with the working conditions in the new country, a positive change in primary health care or the opportunity to help improve the primary healthcare system in their home country might motivate some of them to return.

#### Recertification

Apart from personal aspects, GPs faced professional difficulties when returning to their home country. On one hand, recertification after a certain period of absence seemed too much of a hassle for some to decide to go back; in addition, some GPs were uncertain whether their specialisation as a GP in another country would be recognised at home.

## Discussion

### Summary

This multi-country study was carried out to explore the driving forces influencing GPs’ migration in Europe. The main reasons for migrating to another country, to stay in the new country, to return, or migrate further were grouped under the following three themes: professional development; personal reasons; and the situation in their home country or organisation of health care. Professional development was mentioned by the participants as the driving force of migration; at the same time, the decision to stay or to leave seemed to be influenced by the personal factors the most.

### Comparison with existing literature

A lot of literature has tried to explain the migration of doctors using the terms push, pull, and plant factors. Better and safer working conditions, smaller workload, higher remuneration, the existence of a national public health strategy, political instability, personal insecurity, and future prospects are just some of the factors for the migration of doctors identified in various studies.^10,12,16,18^ The same reasons were mentioned by the participants of the present study (Supplementary Tables S4 and S5). In this study, the focus was laid more on the phases of migration: migrating, staying, or leaving. In all these phases, the same reasons tended to come back and can be seen simultaneously as a pull, a push, or a plant factor. So, while this study can agree on the use of pull, push, and plant factors to describe reasons to migrate, one should be cautious when describing one reason for migration as *only* a push, a pull, or a plant factor. The terms should be used in the right context.

This study described reasons why GPs, as a specific group within the healthcare workforce, tend to migrate back or migrate further. Gauld *et al* used a similar approach, but only studied the reasons why doctors from the UK did not stay very long in New Zealand after migration. Those who went back to the UK initially migrated for professional development and gaining experience, which was also mentioned by some of the present study participants.^24^ This study also described various factors preventing GPs from going back to their home country. Some of them could be seen as plant factors, while others could be seen more as barriers, such as the difficulty of re-entry after a time abroad, or the fear of facing the same problems that motivated their migration in the first place. The ease of re-entry after being away is briefly mentioned in the study by Sharma *et al*.^25^ Brugha *et al* described that some GPs who wanted to migrate further seemed to be pulled or pushed by the same reasons for they were migrating initially: professional and personal reasons, which was another recognisable phenomenon.^13^ These migrants were called *'backpacker migrants'* by Brugha *et al*; they were always in pursuit of better career opportunities, salaries, or overall conditions, hopping from one country to another. This phenomenon is also seen in the study by Gauld *et al*.^24^

In the present study, there was an overrepresentation of GPs from Eastern Europe compared with Western Europe, which accords with the previously described findings from literature that doctors tend to migrate from lower-income countries to higher income countries.^4,7–10^

While some literature underlines the need for retention strategies to combat the shortage of doctors owing to migration, few studies propose specific strategies. McAleese *et al* stated that developing retention strategies is required in order to stop the need of some countries to actively recruit doctors.^8^ Both Humphries *et al* and Taderera emphasised that understanding the reasons why doctors migrate could eventually contribute to much-needed policy changes in service of more robust retention strategies.^26,27^

Ibrahim *et al* described some pull factors that could result in better retention of expat doctors in the UAE.^28^ These could be described as the earlier mentioned plant factors too. Sharma *et al* briefly addressed the fact that easing re-entry policies in the UK could encourage doctors to come back.^25^ Brugha *et al* also focused on factors influencing the decision to migrate, to stay or to return, focusing more on the specific situation in the Ireland.^13^ Although none of these studies focused on GPs, the same factors were also mentioned in the present study. There are factors contributing to migration and the will to stay in a country instead of returning — mostly personal factors, such as the so-called plant factors and the will to gain experience abroad — which policymakers cannot do anything about. There are, however, pull, push, and even some plant factors — such as a higher remuneration, better career opportunities, easier ways to return after being away for a longer time, and in general an improvement of the healthcare system in a country — that could contribute to the retention of GPs and even the return of emigrated GPs.

### Strengths and limitations

For this study, an open call was made to participate in individual interviews and only 15 GPs agreed to participate. There was an overrepresentation of participants from Eastern European countries and some countries were represented by only one GP. It is not possible to report on the viewpoints of GPs who did not participate in the study. Because of these factors, it is difficult to establish actionable points for the entirety of Europe.

The first two authors involved in interviewing the participants and analysing the data have backgrounds in family medicine and general practice, which helped in understanding experiences and terms used by participants, but could also potentially influence the neutrality. Several participants from Estonia were familiar to one of the researchers and were therefore interviewed by the second author. All of the interviews were analysed by the first two authors, bringing together the complex theme structure. By using the same topic guide and having both interviewers conduct the first interview, the two authors created a similar pattern and approach, minimising possible differences between the interviews.

### Implications for research and practice

This study adds further information on the driving forces of GPs migration in Europe. Migration caused by dissatisfaction with the organisation of health care, especially primary health care and living environment, is something countries could potentially work on in order to retain their GPs. The focus needs to be on improving work conditions, education, career opportunities, and progression in order to be able to retain domestically-trained GPs, as well as on motivating emigrated GPs to come back by eliminating barriers for returning home. Once GPs have migrated and are experiencing satisfying working and living conditions elsewhere, it is more difficult to attract them back. Some countries in Europe rely more and more on international recruitment to combat the GP shortage, which in turn worsens the situation in other countries; as such, a more unified Europe-wide approach to GP shortage and migration is needed to help diminish the discrepancies between the countries, and limit the ‘brain drain’ from Eastern Europe to Western Europe. However, as there is not much focus yet on evidence-based retention strategies for GPs, more research on this topic is needed.

## Data

The dataset relied on in this article is available from the corresponding author on reasonable request.
